# Supplementary material for: Design of PG-Surfactants Bearing Polyacrylamide Polymer Chain to Solubilize Membrane Proteins in a Surfactant-Free Buffer
Source: Int J Mol Sci. 2021 Feb 3;22(4):1524. doi: 10.3390/ijms22041524 (PMC7913505; doi:10.3390/ijms22041524)
Supplement: Supplementary file 1 [file ijms-22-01524-s001.pdf]

# Supporting Information

## Design of the PG-surfactants, bearing Polyacrylamide Polymer Chain, and application to the method to solubilize membrane proteins in a surfactant-free buffer.

**Taro Shimamoto[1], Itsuki Nakakubo[1], Tomoyasu Noji[2], Shuhei Koeda[3], Keisuke Kawakami[4,5], Nobuo Kamiya[4], Toshihisa Mizuno[1,3]***

1 *Department of Life Science and Applied Chemistry, Graduate School of Engineering, Nagoya Institute of Technology, Gokiso-cho, Showa-ku, Nagoya, Aichi 466-8555 (Japan);* toshitcm@nitech.ac.jp

2 *Research Center for Advanced Science & Technology, The University of Tokyo, 4-6-1 Komaba, Meguro-ku, Tokyo 153-8904, Japan*

3 *Department of Nanopharmaceutical Sciences, Graduate School of Engineering, Nagoya Institute of Technology, Gokiso-cho Showa-ku, Nagoya, Aichi 466-8555, Japan*

4 *The OCU Advanced Research Institute for Natural Science & Technology, (OCARINA), Osaka City University, 3-3-138 Sugimoto-cho, Sumiyoshi, Osaka 558-8585 (Japan)*

5 *Present address: Biostructural Mechanism Laboratory, RIKEN SPring-8 Center, 1-1-1 Kouto, Sayo, Hyogo 679-5148 (Japan)*

(a)

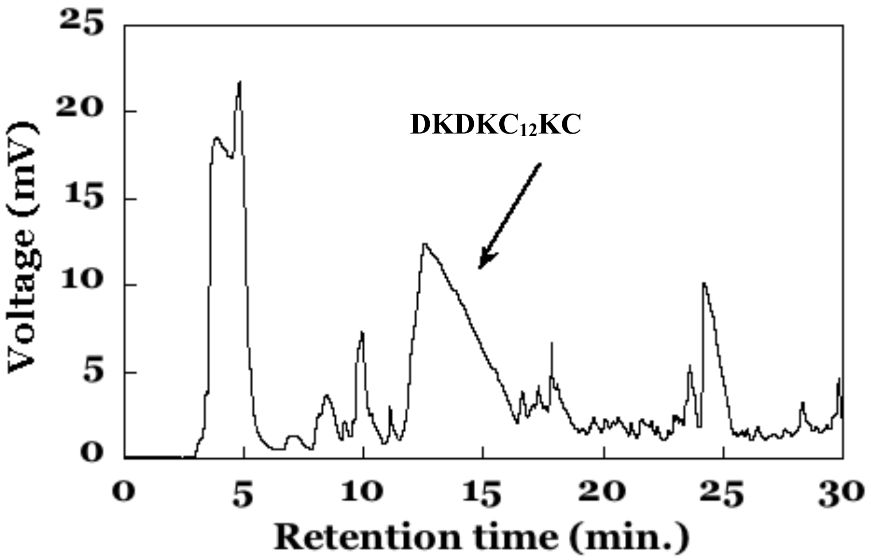


(b)

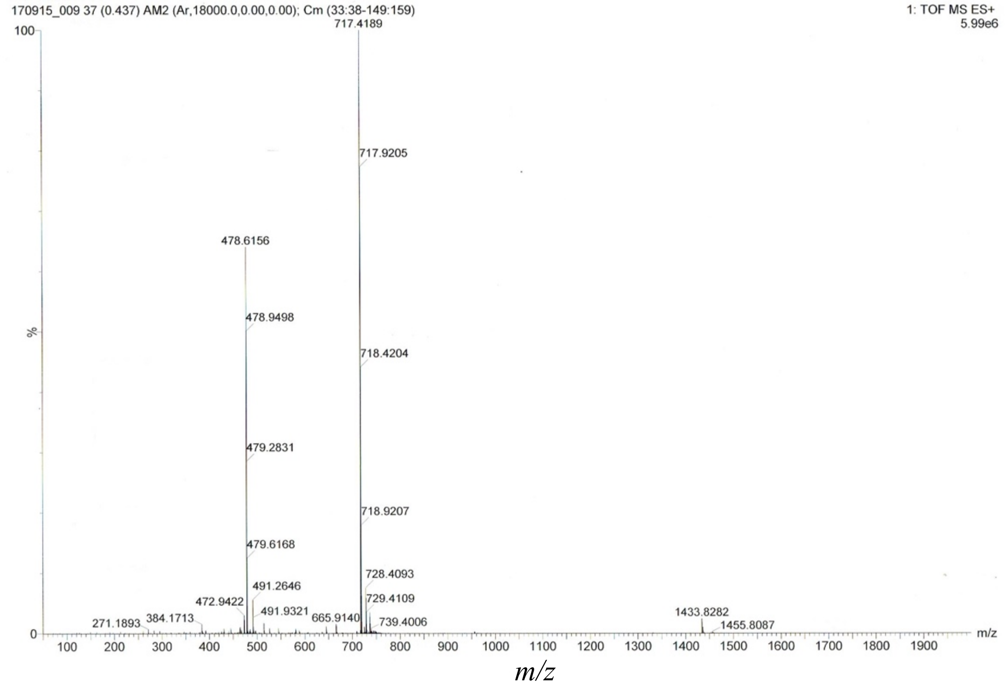


**Figure S1.** Purification of PG-surfactant, **DKDKC$_{12}$KC.** (a) RP-HPLC chromatogram, eluent; linear gradient of MeCN (+ 0.1 vol% TFA)/$H_2O$ (+ 0.1 vol% TFA) from 40/60 to 60/40 for 30 min. (b) High resolution ESI-TOF Mass analysis of **DKDKC$_{12}$KC**.

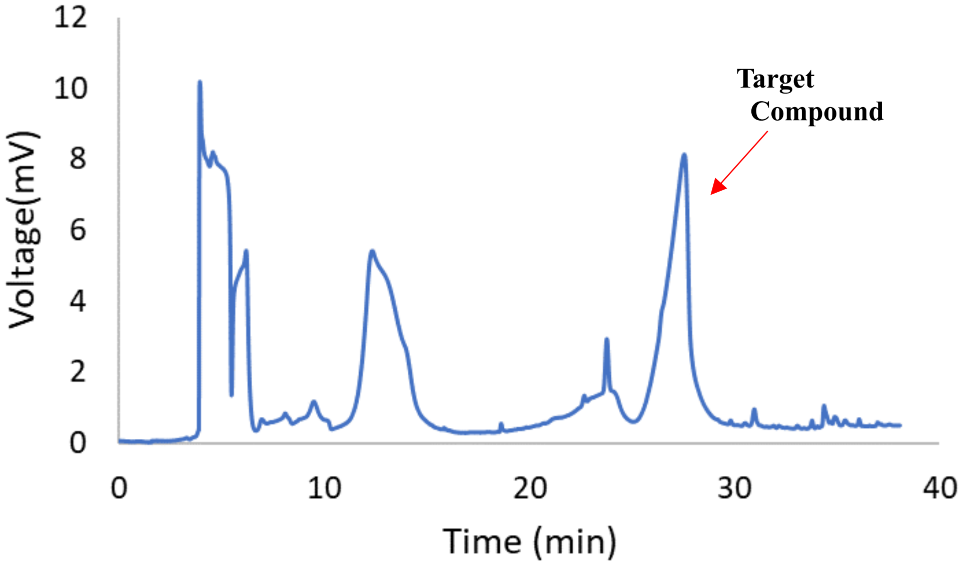


**Figure S2.** Purification of PA-modified PG-surfactant, **DKDKC$_{12}$K-PA$_5$**. Eluent; linear gradient of MeCN (+ 0.1 vol% TFA)/$H_2O$ (+ 0.1 vol% TFA) from 10/90 to 60/40 for 30 min.

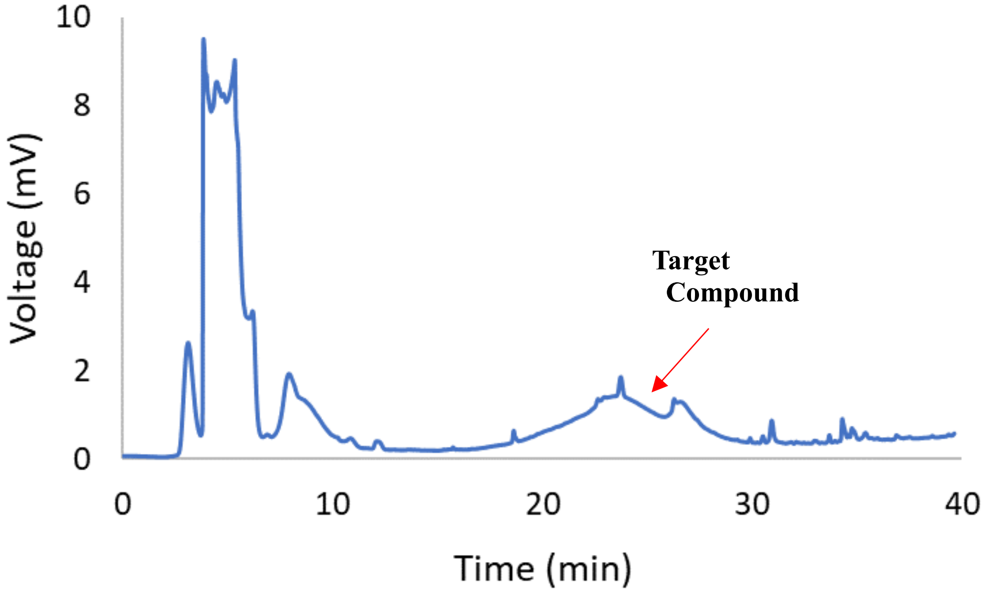


**Figure S3.** Purification of PA-modified PG-surfactant, **DKDKC$_{12}$K-PA$_7$**. Eluent; linear gradient of MeCN (+ 0.1 vol% TFA)/$H_2O$ (+ 0.1 vol% TFA) from 10/90 to 60/40 for 30 min.

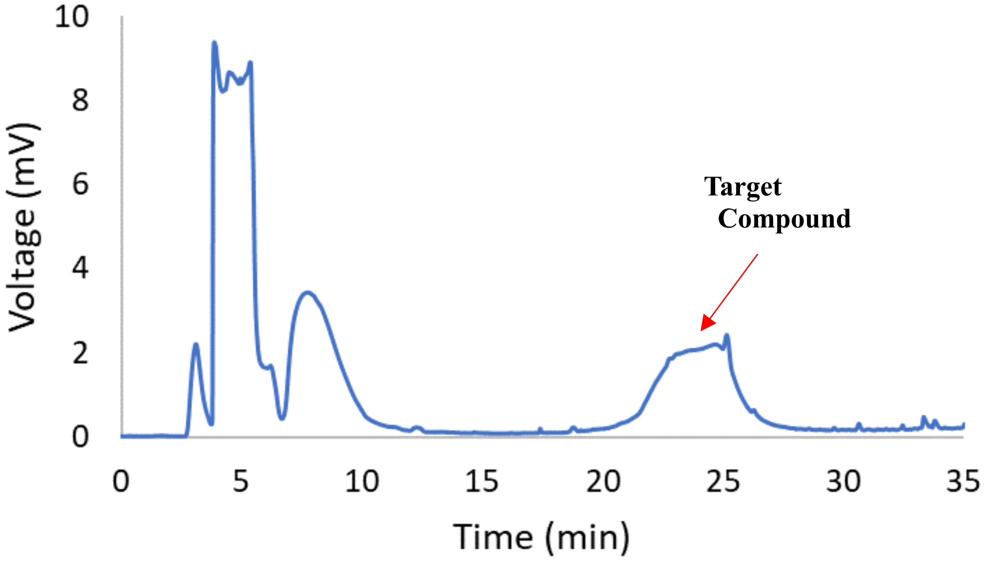


**Figure S4.** Purification of PA-modified PG-surfactant, **DKDKC$_{12}$K-PA$_{18}$**. Eluent; linear gradient of MeCN (+ 0.1 vol% TFA)/$H_2O$ (+ 0.1 vol% TFA) from 10/90 to 60/40 for 30 min.

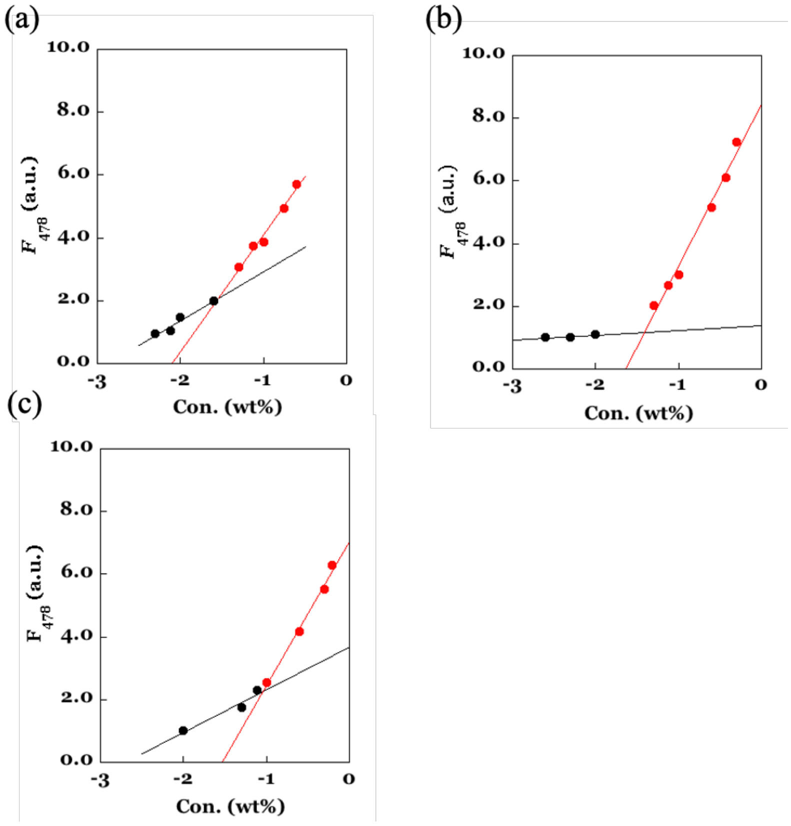


**Figure S5**. Change in $F_{478}$ of 8-anilino-1-naphthalene sulfonate (ANS) in accordance with increased concentration of **DKDKC$_{12}$K-PA$_5$** (a), **DKDKC$_{12}$K-PA$_7$** (b), and **DKDKC$_{12}$K-PA$_{18}$** (c)). [PG-surfactant] = 0–1 mM, [ANS] = 10 µM, 50 mM phosphate buffer, 25 °C.
